# Research progress on risk factors for in-stent restenosis following cerebrovascular stent implantation

**DOI:** 10.3389/fneur.2025.1660202

**Published:** 2025-11-12

**Authors:** Haobo Gao, Hifsa Bibi, Hongtu Tan, Yiwei Zhang, Guofang Yang, Jiabing Wang, Tao Wu

**Affiliations:** 1 Interventional Department, Brain Disease Diagnosis and Treatment Center, The First Affiliated Hospital of Henan University of Chinese Medicine, Zhengzhou, China; 2 Henan Collaborative Innovation Center for the Prevention and Treatment of Major Diseases with Traditional Chinese and Western Medicine, Zhengzhou, China; 3 The First Clinical College of Henan University of Chinese Medicine, Zhengzhou, China

**Keywords:** stents, carotid stenosis, neointima, risk factors, diabetes mellitus

## Abstract

Transient ischemic attack or ischemic stroke within 6 months is frequently associated with severe carotid stenosis. Carotid stent implantation is a widely employed treatment, but in-stent restenosis (ISR) is a dangerous postoperative complication. Many factors cause in-stent restenosis. Previous studies have indicated that stent type, drug use, patient-specific risk factors, levels of various factors in the patient’s body, surgical procedures, and vascular physiological anatomy can all contribute to its occurrence. This review summarizes the key risk factors for ISR following CAS and briefly discusses related findings in intracranial artery stenting, aiming to inform clinical decision-making in neurointerventional practice.

## 1 Introduction

Ischemic stroke is a leading global cause of death, accounting for 5.2% of global mortality. Atherosclerotic carotid stenosis is one of the causes of ischemic stroke in 10–20% of cases (1). Ischemic stroke causes local cerebral ischemia and hypoxia, affects carotid hemodynamics, and induces carotid stenosis (2). Thus, treating carotid stenosis is crucial for curing ischemic stroke. Carotid endarterectomy (CEA) was the main treatment, but not all patients are suitable due to the complex pathogenesis of carotid stenosis. With medical advances, carotid artery stenting (CAS) has emerged as a new method and has achieved remarkable progress (3). CAS restores luminal patency by deploying a stent. Postoperatively, the embolic protection device, catheter, and balloon are removed, while the stent remains to keep the vessel open (4). However, like CEA, CAS also has postoperative complications such as perioperative stroke and postoperative bleeding (5). The occurrence rate of in-stent restenosis (ISR) after CAS is usually 2.0–3.6% (6), Compared to CEA, CAS has a higher incidence of moderate (≥50%) restenosis (7). Among postoperative complications, ISR is seriously impactful. In the International Carotid Stenting Study (ICSS),the 5-year cumulative risk of fatal or disabling stroke after ISR occurrence is 6.4% (8). ISR adversely affects quality of life, increases the likelihood of reintervention, and imposes significant economic burden. A comprehensive understanding of its pathogenesis and risk factors is therefore essential for effective prevention and improved prognosis. In recent years, with medical advances and research progress, understanding of ISR has deepened. Many studies on ISR-inducing risk factors have made significant progress, showing that ISR occurrence is closely related to stent type, patient specific risk factors, and molecular levels (9, 10), while it seems that individual predisposition does not play a crucial role in its pathogenesis (11).

## 2 The definition of CAS and the pathogenesis of ISR

CAS is a cornerstone intervention for carotid stenosis. By deploying balloon catheters and stents, CAS restores luminal patency and improves perfusion to cervico-cerebral territories (12). Nevertheless, post-operative ISR substantially increases the risk of recurrent ischemic stroke and compromises both procedural success and long-term outcome (13). The ISR criteria after percutaneous coronary intervention (PCI) is defined as a vessel lumen restenosis of>50% at the site of the implanted stent, or a stenosis of>50% within the stent or within 5 mm of the stent edge (14). However, most studies on ISR after CAS surgery adopt the ultrasound standard. In one study, a peak systolic velocity (PSV) ≥ 225 cm/s on duplex ultrasound was adopted to define ISR ≥ 50% after carotid artery stenting, whereas a PSV ≥ 300 cm/s or an internal-to-common carotid artery (ICA/CCA) velocity ratio ≥4.0 was used to indicate ISR ≥ 70% (15). Another study has suggested that when using Doppler ultrasound (DUS) for diagnosis, a PSV of 300–330 cm/s, an end-diastolic velocity (EDV) of 120–140 cm/s, or an internal-to-common carotid artery peak systolic velocity ratio (ICA/CCA ratio) between 3.2 and 4.0 typically corresponds to an ISR severity of ≥70% or ≥80% (16). By contrast, a pooled imaging meta-analysis of 5,043 patients with intracranial atherosclerotic stenosis (ICAS) reported an ISR rate of 14.8% over a mean follow-up of 17.8 months. It should be noted that ICAS differs significantly from CAS in terms of vascular anatomy, stent design, and patient risk profiles, and thus these data are presented separately for comparative purposes (17). Angiographic follow-up of 118 patients treated with Enterprise stents for an average of 13.5 months showed a one-year ISR rate of 14.4% (18). The EVA-3S research team, through long-term follow-up of some surgically treated patients, found that during the 3-year follow-up, the cumulative incidence of carotid restenosis in the CAS group was 12.5, 2.3% at 5 years, and 5.0% at 10 years (19). These data underscore the multifactorial nature of ISR and highlight the importance of stent selection according to individual vessel characteristics. Patients with Drug-eluting stents (DES) have a significantly lower ISR rate (3–20%) than those with Bare metal stents (BMS) (20). In addition, patient-specific risk factors can affect ISR occurrence. For example, hyperlipidemia can aggravate lipid deposition in the vessel wall, promote atherosclerotic plaque formation, and induce stenosis (21). At the molecular level, the occurrence of ISR is regulated by various inflammatory factors. They interact through complex signaling pathways, activate the immune system, and thus have an impact (22). Secondly, the vascular anatomical structure, the damage to the vascular wall during stent placement, and the alteration of blood flow patterns after stent placement are all key factors leading to ISR (23). This paper will summarize the latest research progress on the causes of ISR after CAS.

## 3 The relationship between different stent characteristics and ISR

Stent type, length, diameter, strut thickness, and drug-coating status are critical determinants of both procedural success and subsequent ISR risk (24). The development of vascular stents has experienced three main stages: BMS, DES and biodegradable stents (BRS) (25). First, the stent types explored are BMS and DES. In the early days of interventional therapy, BMS were the first-choice stents. Their main mechanism is to improve vascular stenosis and restore vascular patency through mechanical support. Although conventional BMS are fabricated from stainless-steel or cobalt-chromium alloys, ex-vivo primate shunt studies demonstrate that magnesium-based alloys elicit markedly less platelet and fibrin deposition, implying that stent composition directly modulates thrombogenicity and restenosis risk (26). A clinical study of 22 vertebral artery stenosis patients treated with balloon-expandable bare-metal stents found that 6 developed ISR within 1 year, and another 3 cases were detected at the 3-year follow-up, indicating a high long-term ISR risk of 45% with this therapy (27). Another meta-analysis indicates that compared to first-generation single-layer metal stents, second-generation “mesh stents” show a significantly lower ISR occurrence within 30 days (28).

As research deepens, DES have revolutionized interventional surgery. DES are coated with anti-vascular endothelial cell proliferation drugs. They consist of a metal stent matrix, drug-loaded matrix and drugs. The drug-containing coating serves as an intermediate functional layer between the stent and surrounding tissue. By modulating the physical and chemical properties of the stent surface, it controls drug delivery and release rates, optimizing long-term drug efficacy. While curbing smooth muscle cell proliferation, it may also cause stent corrosion (29, 30). The basic characteristics of different types of stents are shown in Table 1.

**Table 1:** Basic characteristics and performance of stent types.

Stent type	ISR incidence	Primary mechanism	Material composition
BMS	28.3% (24.5–32.4%)	Mechanical scaffolding	Stainless steel/ cobalt-chromium alloys
DES	8.7% (6.9–10.8%)	Antiproliferative drug elution	Metallic backbone + permanent or biodegradable polymer
BRS	11.2% (7.4–16.0%)	Biodegradation + controlled drug release	PLLA, Mg/Zn/Fe-based alloys

ISR, in-stent restenosis; BMS, bare metal stents; DES, drug-eluting stents; BRS, biodegradable stents; PLLA, poly-L-lactic acid.

DES have addressed the elastic recoil and neointimal hyperplasia issues seen with BMS to some extent, thus improving treatment effectiveness and reducing ISR occurrence (31). In a retrospective study of patients with symptomatic severe intracranial atherosclerotic disease, 30 patients (31 arteries) treated with DCB between September 2016 and September 2017 were included. Follow-up vascular imaging at 7.0 ± 1.1 months revealed angiographic asymptomatic restenosis in only 3.2% of arteries. These data support the effectiveness of DES in preventing ISR (32). Drug coating is the core of DES. Common antiproliferative agents in coatings are Sirolimus and Paclitaxel. First-generation DES are mainly divided into sirolimus-eluting and paclitaxel-eluting stents (33). In a comparative meta-analysis of two stents’ clinical effects, no significant difference was found between them regarding definite and probable stent thrombus. Long-term follow-up (1–2 and 1–5 years) also revealed no significant differences, indicating they are equally effective in treating stent thrombus (34). In stent implantation, BMS and DES use showed a significant difference in ISR occurrence and rate. Li (35) conducted a single-center prospective cohort study. They gathered 137 patients who underwent vertebral artery ostium (VAO) stent implantation within 1.5 years, with 76 DES and 74 BMS implanted. After an average 12.3-month follow-up, they found that compared with BMS, DES was related to a significantly lower ISR rate. Another single-center retrospective study analyzed 35 patients with stent implantation. It found a 23% ISR rate, a 20% recurrent clinical symptom rate, and that the restenosis rate of DES was significantly lower than that of BMS (36). Given this advantage, to further enhance clinical efficacy and reduce late complications, optimizing the design of drug-eluting coatings and drug release kinetics is currently the core focus of research. In a clinical randomized controlled trial involving 113 patients, it was found that patients receiving the Osstem Cardiotec Centum DES had a significantly lower ISR rate compared to those using the Xience Alpine DES. This suggests that the innovative structural design and drug release mechanism of the new-generation drug-eluting stents contribute to their superiority in reducing ISR and thrombosis risks (37). DES were mainly developed to address the issue of restenosis after stent implantation. However, a new problem emerged: late stent thrombosis, which is thought to be associated with the degradation of the drug carrier on the stent. Therefore, BRS that have both drug-releasing and biocompatible properties are widely considered as the next-generation mainstream stents (38). The research and development of third-generation vascular stents primarily focuses on BRS represented by polylactic acid. Studies on magnesium-based, iron-based, and zinc-based metal stents have made significant progress. During the degradation process, these stent materials can release ions beneficial to blood vessels. For example, biodegradable iron-based stents release ferrous ions during degradation, which can inhibit smooth muscle cell proliferation and thereby reduce the risk of vascular restenosis (39, 40). Atherosclerosis can be seen above, BRS have certain advantages in preventing restenosis. However, the mechanical properties, degradation rate and biosafety of stent materials, as well as achieving an individualized balance between stent and endothelial repair at different lesion sites, are still challenges to be overcome (41). BRS are typically made of polylactic acid and can be completely degraded in the body, eliminating the need for secondary removal surgery. However, the lactic acid generated from Poly-L-lactic acid (PLLA) degradation may cause inflammatory responses, thereby inducing ISR and thrombus formation (42). To address this challenge, Baek coated everolimus (EVL) and surface-modified magnesium hydroxide (mMH) onto BRS. Both in-vitro and in-vivo experiments showed the BRS/EVL/mMH group had better blood compatibility, stronger inhibition of smooth muscle cell proliferation, and better protection of endothelial cell migration and proliferation. Optical Coherence Tomography (OCT) revealed a much lower ISR area than the control group (21%vs87and63%). Also, the BVS/EVL/mMH group had minimal inflammation and thrombosis, with significantly reduced smooth muscle cell proliferation markers and platelet counts. This study offers new thoughts for the clinical use of BMS and BRS (43).

It is important to note that while the biological effects of the type of stent are predominant, deviations in stent size selection can still influence the risk of ISR. Stent length is an independent risk factor for ISR following stent implantation (44). The length of the stent needs to be accurately chosen based on the specific condition of the diseased vessel. Insufficient length can leave the lesion uncovered. The vascular intima at the residual lesion site is still prone to hyperplasia, which may trigger ISR. Stents with a smaller diameter (under 3.5 mm) are connected with a higher ISR risk. But a stent that’s too wide may compress the vessel too much, impacting the normal physiological function of the vascular tissue (45). In addition, stent thickness is also an independent risk factor for ISR (46). Thicker stents have a better supportive force to maintain vascular patency. However, if the stent is too thick, it increases mechanical irritation to the vascular wall, triggering a more intense inflammatory reaction and repair process, thus increasing the risk of ISR. On the other hand, thinner stents may fail to effectively resist forces like vascular elastic recoil, leading to vascular restenosis. Therefore, when evaluating the impact of stents on ISR, both stent type and size are closely related and important factors. In clinical practice, the appropriate stent type must be selected based on lesion characteristics, and optimal stent implantation must be achieved through precise quantification-based treatment. The clinical safety and performance of different stent types are shown in Table 2.

**Table 2:** Clinical performance and biocompatibility of stent types.

Dimension	BMS	DES	BRS
Advantages	High radial strength	Significant ISR reduction	Avoids permanent implant
Limitations	Highest ISR risk	Slightly increased late stent thrombosis	Lower radial strength
Endothelialization time	1–3 months	6–12 months	3–6 months
Biocompatibility	Chronic foreign-body reaction with metal-ion release	Complete resorption, yet lactate or metal-ion release may cause localized inflammatory response	Polymer residues and potential inflammation

ISR, in-stent restenosis; BMS, bare metal stents; DES, drug-eluting stents; BRS, biodegradable stents; PLLA, poly-L-lactic acid.

## 4 The impact of patient-individual factors

After carotid stent implantation, patient-specific factors influence ISR occurrence, being strongly related to gender, metabolic disorders, and unhealthy lifestyle factors. Atherosclerosis is a key mechanism behind poor long-term prognosis post-stenting and ISR development. Hormonal and physiological differences alter endothelial repair and smooth-muscle-cell responses, thereby modifying atherogenesis and ISR propensity. Men, who exhibit more rapid plaque progression, carry a higher ISR burden. Post-menopausal women, deprived of estrogen-mediated vascular protection, require individualized risk stratification (47). Atherosclerosis, the leading cause of ISR, can often be assessed by Intima-Media Thickness (IMT). A multivariate analysis from the Gutenberg Health Study (GHS) showed that sex and age are positively correlated with IMT. In different age groups, males have a significantly higher proportion of carotid plaque than females, and the prevalence is positively correlated with increasing age. The sex difference is most prominent in the oldest age group (65–74 years), where 75.6% of males and 57.7% of females have carotid plaque. This indicates that males have a higher incidence of carotid plaque than females at an early stage (48). Sex hormones are the key to causing this. In males, testosterone levels are positively related to high-density lipoprotein (HDL) and negatively correlated with low-density lipoprotein (LDL) and triglycerides, which cause atherosclerosis. So, low testosterone levels can lead to more blood vessel diseases in males. In females, normal levels of estrogen protect blood vessels. If females have early menopause or their ovaries are removed, leading to a lack of estrogen, they are more likely to have blood vessel diseases (49). Cross-sectional work indicates that IMT integrates traditional risk factors and local hemodynamic forces; consequently, structural vascular changes must be incorporated into any ISR risk model (50). Moreover, IMT is linked to serum uric acid (SUA). SUA, a product of purine metabolism, acts as an inflammatory mediator, inducing endothelial dysfunction and stimulating smooth muscle cell proliferation, making it an independent risk factor for vascular events. SUA has a more significant impact on atherosclerosis in females than in males. Also, females have a lower age threshold than males for the association between SUA and IMT. While elevated SUA levels in both sexes increase the risk of IMT thickening, in peri menopausal females (4–60 years old or≥60 years old), SUA is a more pronounced trigger for changes in hormone levels (51).

Diabetes is characterized by chronic hyperglycemia resulting from insufficient insulin secretion, insulin action defects, or their combination. Recent studies have shown that hyperglycemia can lead to physiological changes through multiple mechanisms, including oxidative stress, inflammatory responses, endothelial dysfunction, and insulin resistance (52). These changes involve the formation of LDL and advanced glycation end products (AGEs), as well as the activation of various pro-inflammatory molecules affecting arterial wall cell types. Consequently, these processes promote neointimal hyperplasia and vascular remodeling, accelerating the progression of atherosclerotic lesions and creating a vicious cycle of “metabolism-inflammation-vessel damage” (53). Both type 1 and type 2 diabetes have been proven to be independent risk factors for accelerating the development of atherosclerosis, and diabetic patients have a 3.47-fold higher risk of ISR than non-diabetic patients (54, 55). A cross-sectional observational cohort study of 187 patients with type 2 diabetes mellitus (T2DM) found a significantly higher prevalence of carotid plaque in the diabetic group than in the control group. This indicates that age, sex, and hypercholesterolemia are positively correlated with carotid plaque formation, further confirming that metabolic abnormalities caused by diabetes can promote the occurrence of ISR (56). In another cross-sectional study of 441 patients with T2DM, C-reactive protein (CRP) was associated with increased carotid IMT in patients with hypertension, and diabetic retinopathy was the only chronic microvascular complication independently associated with advanced carotid atherosclerosis (57). In addition, in T2DM, in males only, the LDL-C/HDL-C ratio is associated with early atherosclerotic vascular structural and functional changes, and is positively correlated with carotid atherosclerosis. This association is not found in female patients. Therefore, gender differences should be considered in the analysis (58).

Cigarette smoking is a well-established independent predictor of ISR. In a long-term follow-up study of 189 patients who underwent CAS, current smokers exhibited a markedly higher ISR risk, underscoring the potential contribution of smoking to post-operative vascular remodeling and restenosis. The underlying mechanisms encompass smoking-induced oxidative stress, endothelial dysfunction, heightened inflammatory responses, and dysregulated lipid metabolism (59). Radiation is also a critical independent risk factor that markedly increases the likelihood of restenosis (60). It inflicts endothelial injury, accelerates atherosclerosis, and induces mural fibrosis (61). Post-radiation carotid stenoses tend to be longer, more diffuse, and atypically distributed, creating a substrate that favors exaggerated neointimal hyperplasia and subsequent restenosis after stent deployment (62).

## 5 The impact of ISR molecular mechanisms

In essence, ISR is the vascular wall’s exaggerated repair response to mechanical injury, involving multiple mechanisms like endothelial cell dysfunction, abnormal Vascular Smooth Muscle Cells (VSMCs) proliferation, and ongoing inflammation (63). In recent years, as research into molecular biology and cellular signaling pathways deepens, the crucial roles of inflammatory molecules and immune cells in ISR development have been increasingly uncovered. Under high-glucose conditions, chronic inflammation is associated with the activation of Toll-like receptor 4 (TLR4). Macrophage migration inhibitory factor 2 (MD2) shows high expression levels. In macrophages, the two combine to form an MD2-TLR4 complex. AGEs bind to MD2, activating the MD2-TLR4 signaling pathway and forming an AGE-MD2-TLR4 complex. This induces the expression of inflammatory factors TNF-*α* and IL-6, and activates the MAPK signaling pathway. Meanwhile, the AGEs/RAGE (Receptor for Advanced Glycation End products) axis also triggers inflammatory responses by activating various downstream signaling pathways such as MAPK, p38, JNK, and JAK/STAT (64, 65). Collectively, these findings indicate a high-glucose environment exacerbates vascular lesions by activating downstream signaling pathways and causes immune cell aggregation, leading to ISR after stent implantation. Moreover, the chronic inflammatory state in diabetics makes blood vessels prone to damage (66). Vascular injury caused by stents activates the immune system, and the abnormal proliferation of VSMCs constitutes the pathological basis of ISR. Single-cell transcriptomics studied the changes in the immune system of tissues near stents after implantation, and found a remarkable M1/M2 polarization imbalance in the macrophage population in the corresponding area (67). M1 macrophages have a relative advantage and secrete cytokines. TGF-β1 can bind to the corresponding receptors on the surface of VSMCs, activate downstream signaling pathways, and induce the transformation of VSMCs from a normal contractile phenotype to a synthetic phenotype. Synthetic phenotype VSMCs have a stronger ability to proliferate, migrate, and synthesize extracellular matrix (ECM), contributes significantly to ISR pathogenesis (68, 69). Moreover, Platelet-Derived Growth Factor-BB secreted by M1 macrophages interacts with Platelet-Derived Growth Factor receptors on VSMCs, promoting a positive-feedback VSMCs phenotypic switch. This causes extensive cell aggregation around the stent, thereby affecting normal vascular structure and function (70). M2 macrophages mainly function by activating the CCL2/CCR2 signaling axis. As a chemokine, CCL2 can specifically bind to the CCR2 receptor on the surface of fibroblasts. This binding sends signals to fibroblasts for directional migration and induces their differentiation into myofibroblasts (71, 72). Myofibroblasts have abundant ECM-synthesis capacity, especially secreting collagen, increasing ECM deposition and altering tissue structure. After scaffold implantation, this can cause adverse reactions like vascular wall thickening and lumen stenosis, impairing vascular patency and function. Imbalanced M1/M2 macrophage polarization and subsequent cellular behavior changes profoundly impact ISR development.

High-mobility group box 1 (HMGB1) acts as a damage-associated molecular pattern (DAMP) by binding to the RAGE, thereby activating histone deacetylases (HDACs). This process leads to an increase in the acetylation level of histone H3K27 in the promoter regions of proliferative genes such as Cyclin D1 and MMP-9, consequently promoting the migration of VSMCs and inducing the occurrence of ISR (73). After vascular injury and exposure, abnormal protein metabolism is triggered, leading to the deposition of a large amount of ECM components such as fibronectin and laminin. This alters the inherent properties of the vascular wall. Additionally, the balance between matrix metalloproteinases (MMPs) and their tissue inhibitors (TIMPs) is disrupted, and the ECM remodeling process is affected by this state of imbalance (74). Oxidative stress-induced reactive oxygen species generation during ferroptosis activates the NF-κB signaling pathway, directly promoting the inflammatory response and abnormal proliferation of VSMCs (75). Recent studies have revealed that vascular structural and functional changes induced by lesions after stent implantation can lead to a local hypoxic microenvironment. This impedes HIF-1α degradation, allowing non-degraded HIF-1α to enter the nucleus. Here, it binds with HIF-1β to form the HIF-1 complex, subsequently initiating the transcription of downstream target genes (76). HIF-1α can also bind to the hypoxia response element on the Hexokinase 2 (HK2) gene promoter, upregulating the expression of HK2 and Lactate Dehydrogenase A (LDHA). This promotes the metabolism and proliferation of VSMCs, as well as the synthesis of the ECM, thereby accelerating the progression of vascular lesions (77). SIRT3 regulates the activity of HIF-1α through deacetylation, which inhibits the upregulation of HIF-1α in the expression of HK2 and LDHA, playing a negative regulatory role (78). Current preventive and therapeutic strategies for patients with diabetes mellitus involve strict pre-operative glycemic control in accordance with the ADA/EASD consensus (target HbA1c ≤ 7.0%), combined with high-intensity statin therapy and PCSK9 inhibitors aimed at achieving an LDL-C level <1.4 mmol/L (ESC 2021, very-high-risk patients), when acute coronary syndrome or multivessel disease is present, the target may be lowered to <1.0 mmol/L. Intra-operatively, drug-eluting stents are preferentially employed.

## 6 The correlation between stent specifications, vascular structure, and ISR

ISR following CAS is related to surgical procedures, hemodynamic factors, and vascular anatomical features. The accuracy of surgical procedures impacts vessel interface integrity, and whether the balloon is sufficiently expanded during the operation also influences postsurgical outcomes. Wall thickness and arterial elasticity modulate stent-vessel interactions. When apposition is complete, wall tension remains physiological, endothelial trauma is limited, and laminar flow is preserved—collectively lowering ISR likelihood (79). If there is stent under-expansion with malposition or overstretching, it will cause reduced vascular wall tension, abnormal blood flow velocity, and turbulent local blood flow, among other pathological conditions. Then, the vascular wall shear stress (WSS) will deviate from normal levels, increasing the risk of post-operative complications and ISR [OCT criteria for incomplete lesion coverage: Axial separation distance between the stent beam and the vascular wall >160 μm (Cypher Select, Cordis, Johnson and Johnson Co., Miami Lake, FL, United States), >130 μm (Taxus Liberte, Boston Scientific, Natick, MA, United States), >110 μm (Endeavor, Medtronic AVE, Santa Rosa, CA, United States), >90 μm (CoStar, Conor Medsystems, Inc., Hamilton, Court Menlo Park, CA, United States)]. Wasser and others studied the relationship between stent length, width, and ISR. Among 210 patients who underwent surgery, they found that for each 1 mm increase in stent length, the risk of ISR rose by 25%; for each 1 mm decrease in stent width, the ISR risk went up by 72%. Longer, narrower stents heighten ISR probability—likely because longitudinal coverage amplifies endothelial injury and radial recoil (80). In addition, stent malpositioning and other procedural errors can result in incomplete lesion coverage, thereby aggravating local blood flow disturbance. There is a relationship between hemodynamic disturbance and the response to carotid sinus stimulation. During stent release, traction on surrounding vessels can stimulate the carotid sinus. According to the NASCET standard, a residual diameter stenosis of ≥30% indicated by postoperative Digital Subtraction Angiography (DSA) or Computed Tomography Angiography (CTA) is defined as residual stenosis (81). Furthermore, a high residual stenosis rate after surgery reflects inadequate expansion, and stress concentration further stimulates endothelial dysfunction (82). When the carotid body is injured, leading to an effect on the baroreceptors, or when blood flow changes exceed the regulatory range of the vascular smooth muscle, cerebral autoregulation is impaired. In such cases, hyperperfusion syndrome is likely to occur (83). Stent implantation alters the geometric configuration and hemodynamic characteristics of blood vessels. In particular, turbulent flow, vortices, and low-velocity regions tend to form at the edges and curves of stents. These abnormal blood flow patterns can damage endothelial cells and reduce local WSS. Excessive blood flow increases shear stress, leading to endothelial injury. On the other hand, excessively low WSS reduces the compressive force of the stent on the vascular wall. This affects vascular remodeling and leads to the occurrence of ISR (84). Abnormal blood flow velocity and pressure affect the mechanical properties of vascular walls. Fast blood flow increases WSS, causing endothelial damage, while low blood flow pressure reduces the stent’s compressive force on the vascular wall, hindering vascular remodeling. Both factors increase the risk of ISR. Stent implantation changes vascular hemodynamics, influencing carotid plaque formation and local vascular structural remodeling. Another study using computational fluid dynamics analyzed the hemodynamic changes after stent implantation, finding that stent length significantly impacts local blood flow velocity and WSS. When the stent protrudes 1 mm beyond the vascular wall, the decrease in blood flow velocity and WSS is minimized, lowering the risk of thrombosis and neointimal hyperplasia, and consequently decreasing ISR occurrence (85). When the vessel diameter is <4.5 mm, the ISR rate is 36%; whereas for patients with a vessel diameter >4.5 mm, the ISR rate is only 12%. This may be because slender and long vessels typically generate stronger elastic recoil (86). A retrospective analysis of 931 carotid stenosis patients who received treatment evaluated the pre-and post-operative (average 12-month follow-up) carotid vascular structure characteristics via color Doppler flow imaging (CDFI). It found that a post-operative distal common carotid artery diameter<6.8 mm and a post-operative ratio of the bulb to the distal common carotid artery diameter>1.0 were both independent risk factors for post-operative ISR. The carotid bifurcation has a special configuration, which can be divided into a “Y”-shaped bifurcation, a “tuning-fork” -shaped bifurcation, and a “ladle”-shaped bifurcation. These different types have varying effects on hemodynamic instability and vulnerability, for instance, tortuous vessels and angular sites. Its natural blood flow stratification is no longer synergistic with the compliance lost after stent implantation, thus facilitating intimal hyperplasia and thrombus formation. Local hemodynamics and flow patterns vary with the vascular angle. The larger the angle, the higher the WSS. Both low (<0.4 Pa)and high (>40 Pa)WSS promote ISR through distinct mechanisms (87). At the bifurcation, the blood flow dynamics feature localized low endothelial shear stress on the lateral walls of the main and branch vessels, predisposing these regions to atherosclerotic plaque formation. At the bifurcation, plaque morphology may present as eccentric or concentric plaque. The distribution of these irregular plaques further exacerbates blood flow turbulence and elevates the risk of thrombosis. Meanwhile, stent implantation disrupts the original hemodynamic equilibrium, inducing neointimal hyperplasia and vascular remodeling (88). Furthermore, cone-beam CT (CBCT) detection of the internal carotid artery (ICA) in 161 patients with intracranial calcification revealed an increased incidence of calcification in the C1, C5/C6, and C4 segments. The calcification rates in these segments were found to increase with age and exhibited certain gender differences. Specifically, the rates of moderate and severe calcification in the C1, C4, and C5/C6 segments were higher in males than in females (89). In patients with anatomically complex vasculature (tortuosity angle >70° or vessel diameter <4.5 mm), the preventive and therapeutic approach consists of using shorter stents to reduce overall coverage length and selecting pre-dilatation balloons of a 1:1 diameter ratio to the vessel to avoid over-expansion. Computational fluid dynamics is employed to assess WSS, and rotational atherectomy or shock-wave balloon pre-treatment is performed for severe calcification to achieve a residual stenosis <30%. Post-operative surveillance is conducted at 1, 3, and 6 months with duplex ultrasonography or magnetic resonance angiography; if a peak systolic velocity ≥230 cm/s or ≥50% restenosis is detected, prompt re-intervention is initiated.

## 7 Conclusion and future perspectives

Rapid advances in neuro-interventional techniques have expanded our understanding of in-stent restenosis (ISR), yet its prevention and management remain a central challenge in cerebrovascular therapy. This paper systematically reviews the key risk factors for ISR occurrence, from the characteristics and mechanical properties of stent materials to individual patient risk factors and pathogenesis, and provides a comprehensive overview of the etiology of ISR development. Looking to the future, more in-depth research should be conducted in the following directions: (1) Further optimization of stent design and material development: Development of new types of stents with superior biocompatibility and precise regulation of drug release in response to changes in the vascular physiological microenvironment, in order to minimize interference with normal vascular physiological functions after stent implantation and fundamentally reduce the incidence of ISR; (2) Molecular targeted intervention strategies: Integrating genomics, transcriptomics, proteomics, and metabolomics will refine ISR biomarker panels and reveal druggable nodes within inflammatory, proliferative, and metabolic pathways; (3) Innovation in dynamic monitoring technologies: Exploration of the application value of new imaging techniques and biosensors in ISR diagnosis, improving the detection accuracy and sensitivity of intravascular microstructure and microenvironment changes, enabling doctors to make accurate diagnoses and adjust treatment plans in a timely manner in the early stages of ISR. (4) To construct a robust research framework for post-CAS ISR, future efforts should integrate both in-vitro and in-vivo basic science studies. In-vitro work can exploit microfluidic platforms that recapitulate carotid anatomy and hemodynamics to dissect how stent-based drug-elution kinetics and immune-cell crosstalk jointly govern endothelial and smooth-muscle-cell behavior. In-vivo studies require standardized large-animal CAS models coupled with multimodal imaging to longitudinally track neointimal hyperplasia, stent degradation profiles and ISR acceleration by comorbidities. This dual approach may overcome the current limitations of ISR research. The future challenges of cerebrovascular interventional therapy lie not only in broadening the indications for surgery, but also in optimizing perioperative management, establishing more efficient emergency pathways, and through device innovation plus the rational concomitant use of antiplatelet, thrombolytic, and anti-inflammatory agents improving recanalization rates and clinical outcomes, while concurrently exploring cell protective strategies for reperfusion. In recent years, artificial intelligence aided assessment systems have markedly elevated the diagnostic and evaluative standards for cerebrovascular disease, offering more effective support for clinical decision making; their scope encompasses ASPECT scoring, flow diverter stent simulation, hemodynamic parameter evaluation, among others. Simultaneously, hospitals must intensify the cultivation and recruitment of medical personnel, advance physicians professional competence and technical proficiency, propel the development and application of relevant technologies, and investigate their deployment in fields such as stroke emergency care and electronic monitoring. Importantly, research must fully acknowledge the particularities of the neuro-interventional field: the distinctive vascular anatomy and the presence of the blood–brain barrier can modify the pathobiology of ISR, underscoring the need for a research framework separate from that used for coronary ISR. Multicenter, randomized, controlled trials powered for hard neurological endpoints are now essential to validate these mechanistic insights and deliver precision, patient-specific neuro-endovascular therapy.
